# Systematic genetic array analysis links the *Saccharomyces cerevisiae *SAGA/SLIK and NuA4 component Tra1 to multiple cellular processes

**DOI:** 10.1186/1471-2156-9-46

**Published:** 2008-07-10

**Authors:** Stephen MT Hoke, Julie Guzzo, Brenda Andrews, Christopher J Brandl

**Affiliations:** 1 Department of Biochemistry, Schulich School of Medicine & Dentistry, University of Western Ontario, London, N6A 5C1, Canada; 2 Department of Medical Genetics and Microbiology and the Banting and Best Department of Medical Research, Terrence Donnelly Centre for Cellular and Biomolecular Research, University of Toronto, Toronto, M5S 3E1, Canada

## Abstract

**Background:**

Tra1 is an essential 437-kDa component of the *Saccharomyces cerevisiae *SAGA/SLIK and NuA4 histone acetyltransferase complexes. It is a member of a group of key signaling molecules that share a carboxyl-terminal domain related to phosphatidylinositol-3-kinase but unlike many family members, it lacks kinase activity. To identify genetic interactions for *TRA1 *and provide insight into its function we have performed a systematic genetic array analysis (SGA) on *tra1*_*SRR*3413_, an allele that is defective in transcriptional regulation.

**Results:**

The SGA analysis revealed 114 synthetic slow growth/lethal (SSL) interactions for *tra1*_*SRR*3413_. The interacting genes are involved in a range of cellular processes including gene expression, mitochondrial function, and membrane sorting/protein trafficking. In addition many of the genes have roles in the cellular response to stress. A hierarchal cluster analysis revealed that the pattern of SSL interactions for *tra1*_*SRR*3413 _most closely resembles deletions of a group of regulatory GTPases required for membrane sorting/protein trafficking. Consistent with a role for Tra1 in cellular stress, the *tra1*_*SRR*3413 _strain was sensitive to rapamycin. In addition, calcofluor white sensitivity of the strain was enhanced by the protein kinase inhibitor staurosporine, a phenotype shared with the Ada components of the SAGA/SLIK complex. Through analysis of a GFP-Tra1 fusion we show that Tra1 is principally localized to the nucleus.

**Conclusion:**

We have demonstrated a genetic association of Tra1 with nuclear, mitochondrial and membrane processes. The identity of the SSL genes also connects Tra1 with cellular stress, a result confirmed by the sensitivity of the *tra1*_*SRR*3413 _strain to a variety of stress conditions. Based upon the nuclear localization of GFP-Tra1 and the finding that deletion of the Ada components of the SAGA complex result in similar phenotypes as *tra1*_*SRR*3413_, we suggest that the effects of *tra1*_*SRR*3413 _are mediated, at least in part, through its role in the SAGA complex.

## Background

*TRA1 *is an essential gene in *Saccharomyces cerevisiae *that encodes a 437 kDa protein product. It is a member of a family of key signaling and regulatory molecules that contain a C-terminal phosphatidylinositol-3-kinase (PI3K) domain [1] and is a component of two multisubunit transcriptional regulatory complexes, the SAGA/SLIK and NuA4 complexes, which also contain the histone acetyltransferase enzymes, Gcn5 and Esa1, respectively [2-4]. Tra1 interacts directly with transcriptional activator proteins and is thought to be critical in recruitment of SAGA/SLIK and NuA4 to their target promoters [5-8].

Previously we identified mutations in the C-terminal PI3K domain of Tra1 that showed defects in transcriptional activation, sensitivity to ethanol and the cell wall destabilizing agent calcofluor white and resulted in shortened telomeres [9]. The pattern of changes neither fully mimicked those seen upon disruption of other SAGA/SLIK nor NuA4 components. For example, unlike strains with deletions of NuA4 components, the *tra1 *mutant strains were relatively insensitive to DNA damaging agents. We performed an initial systematic genetic array (SGA) analysis with the most pronounced allele, *tra1*_*SRR*3413_. This analysis did not identify any synthetic lethal interactions but did reveal 23 synthetic slow growth interactions, many in combination with deletions of genes involved in cell membrane/wall processes. As the lack of synthetic lethal interactions may have arisen from an incomplete selection against diploids in the automated screens, we repeated the screen in a strain background that selects more strongly against diploids. This analysis identified 114 genes displaying synthetic sick/lethal (SSL) interactions with *tra1*_*SRR*3413_. Genes involved in transcription, RNA processing, mitochondrial function and membrane sorting/protein trafficking were prevalent. The phenotypes and genetic interactions of these strains also point to a role for Tra1 in the cellular response to stress.

## Results

### SGA analysis of tra1_*SRR*3413_

As a component of both SAGA and NuA4 complexes, Tra1 is positioned to play a major role in nuclear processes. Previously we performed an SGA analysis on Tra1 using an allele that partially impairs function [9]. No synthetic lethal interactions were obtained in this analysis. We thus chose to repeat the study using a strain that more strongly selects against the spurious appearance of diploids. *tra1*_*SRR*3413 _was integrated into yeast strain BY7092 and SGA analysis performed using the collection of nonessential yeast knockout strains. Haploids were analyzed on synthetic complete (SC) media at 26°C, 34°C and 36°C with pinnings performed in quadruplicate. 224 double mutant strains, scored as having potential synthetic interactions in each of the screens, were manually tested for growth on YPD media at 30°C and SC media at 33.5°C, after sporulation of the diploids and germination of spore colonies on YPD. For each strain comparisons were made to the relevant single disruption strain. As shown in Table 1, 114 genetic interactions were confirmed as either synthetic lethal or synthetic slow growth on SC or YPD media (SSL interactions). Identified genes are organized according to similarities in associated gene ontology terms. Many cellular functions are represented but the most prominent group were genes linked to membrane sorting/protein trafficking with an emphasis on vacuolar function. An overlapping group included genes associated with cell wall biogenesis and function. Other groups identified initially were chromosomal functions, RNA processing, gene expression, metabolism and biosynthesis and mitochondrial function. A clear subgroup of a larger chromosomal functions group contained the gene encoding the alternative histone H2AZ (*HTZ1*) and members of the SWR1 complex, which exchange H2AZ for histone H2A within nucleosomes (for example see [10]).

**Table 1:** Genes with synthetic slow growth or synthetic lethal interactions with *tra1*_*SRR*3413_

***GENE*^1^**	**Orf**	**SSL^2^**	**GO^3^**	**Location, component^4^**	**phenotype^5^**	**stress^6^**	**#^7^**
*MCK1*	YNL307C	SL	1, 3	cytoplasm, nucleus		X	28
*CDC73*	YLR418C	s	1, 3, 10	Cdc73/Paf complex, nucleus	T, R	X	159
*CSE2*	YNR010W	S	1, 3, 10	mediator complex	R	X	19
*HTZ1*	YOL012C	S	1, 3, 10	nucleus			186
*LEO1*	YOR123C	s	1, 3, 10	Cdc73/Paf complex, nucleus	T		39
*RAD52*	YML032C	s	1, 3, 10	nucleus		X	196
*SPT8*	YLR055C	s	1, 3, 10	SAGA complex, nucleus			62
*SRB2*	YHR041C	s	1, 3, 10	Srb-mediator complex	T, E		34
*SWR1*	YDR334W	s	1, 3, 10	SWR1 complex, nucleus	R		112
*VPS71*	YML041C	s	1, 3, 10, 9	SWR1 complex, nucleus	E		97
*VPS72*	YDR485C	s	1, 3, 10, 9	SWR1 complex, nucleus	E		87
*KAR3*	YPR141C	s	1, 3, 7	microtubule, spindle pole body	E		131
*UBI4*	YLL039C	s	1, 3, 8, 9	cytoplasm		X	7
*UBP15*	YMR304W	s	1, 3, 8, 9	cytoplasm			0
*SWC3*	YAL011W	s	1, 3, 9, 10	SWR1 complex, nucleus, mitochondrion			85
*DOA1*	YKL213C	s	1, 9, 8	cytoplasm, nucleus		X	26
*TOR1*	YJR066W	S	1,2,3,4,5,6,7,8,9,10	cellular membranes	R	X	27
*CKB2*	YOR039W	s	1,2,3,4,5,7,8,9,10	protein kinase CK2, CURI complex		X	14
*SNF1*	YDR477W	s	1,3,4,5,6,7,8,9,10	cytoplasm, nucleus, nuclear evelope, mitochondrion, vacuole	R	X	18
*SNF4*	YGL115W	S	1,3,4,5,6,7,8,9,10	cytoplasm, nucleus, nuclear envelope, PM			7
*CNB1*	YKL190W	s	1,3,4,5,7,9,10	calcineurin complex		X	48
*EOS1*	YNL080C	S	1,3,4,7	ER membrane		X	4
*SIS2*	YKR072C	s	1,3,5,10	cytoplasm, nucleus		X	10
*NUM1*	YDR150W	s	1,3,7,9	cell cortex, cellular bud tip, mitochondrion			57
*SWI4*	YER111C	s	1, 3, 7, 10	nucleus	CW		69
*UBP6*	YFR010W	S	1,3,8,9,10	proteosome		X	28
*MSC1*	YML128C	s	1,3,9,6	ER, mitochondrion	R		14
*ARP6*	YLR085C	s	10, 1, 2	SWR1 complex, cytoplasm	R		149

*CAF40*	YNL288W	s	2, 10	CCR4-NOT complex	R		8
*NST1*	YNL091W	s	2, 10	cytoplasm		X	2
*PUB1*	YNL016W	s	2, 10	cytoplasm, nucleus, hnRNP complex		X	5
*PIH1*	YHR034C	s	2, 3, 8, 10	cytoplasm, nucleus, snRNP complex			2
*HMT1*	YBR034C	s	2, 4 10	nucleus			4
*PML39*	YML107C	SL	2, 4, 10	nuclear pore, ribosome			4
*HCR1*	YLR192C	s	2, 8	translation IF3 complex	T	X	5
*PPQ1*	YPL179W	S	2, 8	cytoplasm			0
*TMA23*	YMR269W	s	2, 8	nucleolus, ribosome			5
*RRP6*	YOR001W	SL	2, 8, 10	nuclear exosome (RNase complex)			16
*SKY1*	YMR216C	s	2,3,4,8,10	cytoplasm			5
*STP1*	YDR463W	s	2,4,7,8,10	nucleus			2

*KNS1*	YLL019C	SL	3			X	0
*BIM1*	YER016W	s	3, 1, 7	cytoplasm, microtubule, kinetochore, spindle pole body			282
*NBP2*	YDR162C	s	3, 7	cytoplasm, nucleus	CW, R	X	49
*PRB1*	YEL060C	s	3, 8, 9	vacuole		X	3
*SEL1*	YML013W	s	3, 8, 9	ubiquitin ligase complex, ER, mitochondrion			3
*TPS2*	YDR074W	s	3,5,7,8,10	mitochondrion		X	4

*ITR1*	YDR497C	s	4, 5, 7, 9	plasma membrane		X	9
*NEW1*	YPL226W	s	4, 5, 9, 8	cytoplasm, mitochondrion			8
*AQR2*	YBR043C	s	4, 7	plasma membrane			1
*GTR1*	YML121W	s	4, 9	cytoplasm, endosome, nucleus, vacuole	T, E, R		5
*VAM10*	YOR068C	SL	4, 9	vacuolar membrane			11

*SER1*	YOR184W	s	5	cytoplasm			1
*SER2*	YGR208W	s	5	cytoplasm, nucleus			5
*GCR2*	YNL199C	SL	5, 10, 3	nucleus	E		30
*ARO1*	YDR127W	SL	5, 6	cytoplasm	E		0
*ARO2*	YGL148W	SL	5, 6	cytoplasm			1
*ETR1*	YBR026C	s	5, 6	mitochondrion			0
*LIP2*	YLR239C	s	5, 6	mitochondrion			7
*LIP5*	YOR196C	S	5, 6	mitochondrion			2
*OAR1*	YKL055C	s	5, 6	mitochondrion			0
*ERG3*	YLR056W	s	5, 9	ER			19

*MDM34*	YGL219C	S	6	mitochondrial outer membrane	E, R		10
*KGD2*	YDR148C	S	6, 5	mitochondrion			0
*MDM10*	YAL010C	s	6, 9	Mdm10/Mdm12/Mmm1 complex, mitochondrial outer membrane	T, E		11

*BEM1*	YBR200W	S	7, 3, 1	bud neck	E		33
*BEM4*	YPL161C	s	7, 3, 1	cytoplasm, nucleus	T, E	X	29
*Smi1*	YGR229C	s	7, 3, 1	bud tip, nucleus	T, E, CW, R		111
*ROM2*	YLR371W	SL	7, 3, 9	bud tip		X	16
*CSF1*	YLR087C	s	7, 5, 6	mitochondrion	CW		24
*ECM30*	YLR436C	s	7, 9	cytoplasm	CW		1
*KRE1*	YNL322C	s	7, 9	cell wall			64
*VIP1*	YLR410W	S	7, 9	cytoplasm			15
*TPM1*	YNL079C	S	7,9,3	actin cable	E, CW, R		21

*RPS27B*	YHR021C	S	8	ribosome	T		1
*RPN4*	YDL020C	S	8, 10, 1, 3	nucleus, proteasome	T, E	X	242
*YDJ1*	YNL064C	S	8, 9, 6	cytosol, microsome	R	X	27

*CCZ1*	YBR131W	s	9	late endosome, membrane	E, R	X	6
*MON1*	YGL124C	s	9	cytosol, vacuolar membrane			12
*VAM3*	YOR106W	s	9	vacuolar membrane	R		9
*VAM7*	YGL212W	s	9	vacuolar membrane	T, E, CW, R		8
*PIB2*	YGL023C	s	9, 1, 7	late endosome, mitochondrion	R		0
*GSG1*	YDR108W	s	9, 3	TRAPP complex		X	11
	YDR049W	s	9, 3, 8	cytoplasm, mitochondrion	E, R		
*MEH1*	YKR007W	S	9, 4, 5	cytosol, vacuolar membrane		X	2
*PMR1*	YGL167C	s	9, 4, 7	Golgi membrane	E		41
*AUT7*	YBL078C	s	9, 5	autophagic vacuole, cytosol		X	2
*CCW12*	YLR110C	s	9, 7	cell wall	CW		13
*COG5*	YNL051W	S	9, 7	Golgi			14
*COG6*	YNL041C	S	9, 7	Golgi			17
*COG7*	YGL005C	S	9, 7	Golgi			12
*COG8*	YML071C	S	9, 7	Golgi			17
*DID2*	YKR035W	s	9, 7	cytoplasm, late endosome			2
*GYP1*	YOR070C	S	9, 7	Golgi, mitochondrion	R		48
*MNN10*	YDR245W	s	9, 7	α-1,6-mannosyltransferase complex	E, R, CW		19
*MON2*	YNL297C	s	9, 7	cytosol, endosome	R		40
*RIC1*	YLR039C	S	9, 7	Golgi, nucleus	E		159
*RUD3*	YOR216C	s	9, 7	Golgi			26
*SEC22*	YLR268W	s	9, 7	ER, Golgi	CW		44
*TLG2*	YOL018C	s	9, 7	early endosome, trans Golgi	R		12
*VPS1*	YKR001C	S	9, 7	cytoplasm, membrane fraction			28
*VPS17*	YOR132W	s	9, 7	endosome, retromer complex			11
*VPS38*	YLR360W	s	9, 7	Vps34 complex	R		12
*VPS41*	YDR080W	s	9, 7	HOPS complex, vacuolar membrane, endosome	R		7
*VPS53*	YJL029C	SL	9, 7	GARP complex, Golgi, cytoplasm	T, R		10
*YPT6*	YLR262C	s	9, 7	Golgi	E, R		181
*YPT7*	YML001W	s	9, 7	vacuole, mitochondrion	T, R		6
	YLR111W	s	9, 7		CW		0
*GET1*	YGL020C	S	9, 8	GET complex, ER, mitochondrion	R	X	284
*SSE1*	YPL106C	SL	9, 8	cytoplasm	T		15
*BTS1*	YPL069C	S	9,5,6	mitochondrion	CW		16
*UBP3*	YER151C	s	9,7,10,1,3	cytoplasm	R		32

	YDL180W	s		vacuole			0
	YEL033W	s			T		0
	YOR235W	s					0

^1 ^Genes are placed according to related GO terms with relevant groupings separated by lines.

^2 ^Phenotype of double mutants. SL, synthetic lethal; S, severe slow growth; s, slow growth (in SC media at 33.5°C). For those underlined the phenotype is most easily detected on YPD at 30C as the single mutant shows reduced growth on SC media

^3 ^Gene ontology: (1) DNA replication/repair and chromosome stability, (2) RNA processing, (3) cell cycle/microtubule, (4) general transport, (5) metabolism, (6) mitochondria, (7) polarity/cell wall, (8) protein/ribosome biosynthesis, (9) secretion/protein trafficking, (10) transcription, (11) unknown

^4 ^Cellular compartments and/or components as indicated in the Saccharomyces Genome Database

^5 ^Relevant phenotypes of single mutants as described in the Saccharomyces Genome Database. E, sensitivity to ethanol; CW, sensitivity to calcofluor white; T, shortened telomeres; R, sensitivity to rapamycin

^6 ^Annotated in the Saccharomyces Genome Database as involved in stress response or autophagy

^7 ^Number of previously identified SSL interactions in the Saccharomyces Genome Database

Before pursuing further analysis of the genes identified in the SGA screen, we wanted to eliminate those that may have arisen through indirect effects on neighboring genes. For instance, YLR111W is a dubious ORF located adjacent to *CCW12 *that encodes a cell wall component. Since disruption of YLR111W may simply act by affecting expression of *CCW12*, it was not considered in further analyses. Several other dubious ORFs were eliminated because they overlapped a second identified gene. Other pairs of adjacent genes were *kgd2 *and *num1*, *spt8 *and *erg3*, and *tpm1 *and *eos1*, though these were not removed from the analysis since potentially both could be involved in SSL interactions with *tra1*_*SRR*3413_.

Also indicated in Table 1 is the total number of additional SSL interactions listed for each of the genes in the Saccharomyces Genome Database. It has been argued that the number of interactions may be a measure of the importance of a gene for cellular fitness [11]. The relatively large number of SSL interactions for *tra1*_*SRR*3413 _may also reflect its involvement in both SAGA/SLIK and NuA4 complexes. As with any synthetic lethal analysis we can not eliminate the possibility that some of the apparent interactions are due to additive growth defects rather than a demonstration that the genes act in the same or related pathways.

**Figure 1:**
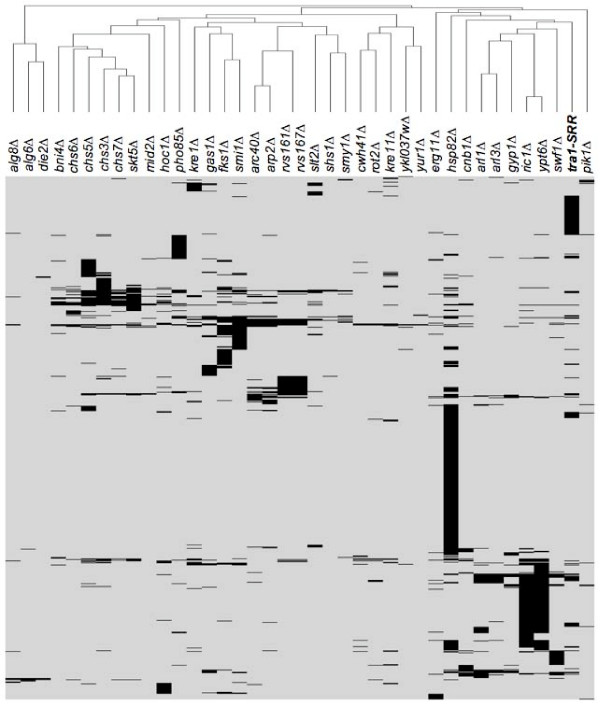
**Hierarchal cluster analysis of *tra1*_*SRR*3413 _SSL interactions**. Agglomerative hierarchical clustering based on the average linkage of uncentered correlations using CLUSTER 3.0 software [50] was performed on the profile obtained with the *tra1*_*SRR*3413 _strain and the combined data sets of Tong et al. [11], Measday et al. [12], Reguly et al. [13], Pan et al. [14], Mitchell et al. [15] and including SSL interactions of additional SAGA/SLIK and NuA4 components as listed in the Saccharomyces Genome Database. Only the profiles clustering close to *tra1*_*SRR*3413 _are shown.

To identify functional relationships for *tra1*_*SRR*3413_, a hierarchal cluster analysis of SSL *tra1*_*SRR*3413 _interactions was performed with the data sets of Tong et al. [11], Measday et al. [12], Reguly et al. [13], Pan et al. [14] and Mitchell et al. [15] plus the SSL interactions of additional SAGA/SLIK and NuA4 components as listed in the Saccharomyces Genome Database (Figure 1). *tra1*_*SRR*3413 _clustered most closely to a group including *arl1Δ0*, *arl3Δ0*, *gyp1Δ0*, *ric1Δ0*, *ypt6Δ0 *and *swf1Δ0*. Three of these (*ric1*, *ypt6 *and *gyp1*) show synthetic interactions with *tra1*_*SRR*3413 _(Table 1). All encode key regulatory molecules in the processes of membrane sorting and protein trafficking [16], and with the exception of *SWF1 *are GTPases of the Ras-family or regulatory proteins of these molecules. Interestingly, *ric1 *was first identified as a temperature sensitive allele that affected transcription of both ribosomal protein genes and rRNA [17]. Also closely associated with this group was *cnb1Δ0*, which is SSL with *tra1*_*SRR*3413 _and encodes a Ca^2+^/calmodulin dependent protein phosphatase required for cell cycle regulation, stress induced gene expression and cell wall synthesis. To gain additional confidence in the apparent association of *tra1*_*SRR*3413 _with the family of GTPases, the cluster analysis was repeated in the absence of these genes. In this case, *tra1*_*SRR*3413 _clustered with a group containing SAGA/SLIK and NuA4 components (not shown).

### Sorbitol partially suppresses defects due to tra1_*SRR*3413_

**Figure 2:**
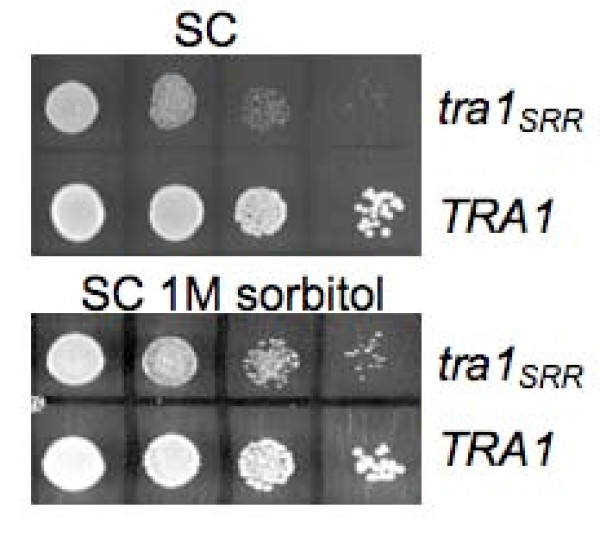
**Sorbitol partially suppresses slow growth at 37°C due to *tra1*_*SRR*3413_**. Yeast strains CY2222 (*tra1*_*SRR*3413_) and BY7092 (*TRA1*) were grown overnight to saturation in YPD at 30°C. Cells were diluted to approximately 1000 cells per μl and 10 μl of 10-fold serial dilutions spotted on synthetic complete media containing 2% glucose without (SC) or with 1.0 M sorbitol. Cells were grown for 4 days at 37°C.

**Figure 3:**
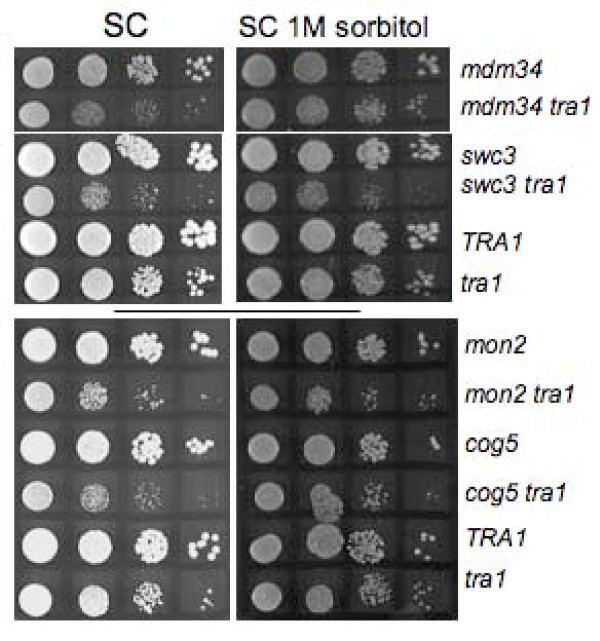
**Growth of *tra1*_*SRR*3413 _double mutant strains in sorbitol**. Strains containing the indicated single disruptions (*mdm34*, *swc3*, *cog5 *and *mon2*) and combinations with *tra1*_*SRR*3413 _(*tra1*) were generated by sporulation of diploids obtained from SGA analysis. These strains plus CY2222 (*tra1*_*SRR*3413_) and BY7092 (*TRA1*) were grown overnight to saturation in YPD at 30°C. Cells were diluted to approximately 2000 cells per μl and 5 μl of 10-fold serial dilutions spotted on synthetic complete media containing 2% glucose without (SC) or with 1.0 M sorbitol. Cells were grown for 3 days (upper grouping) or 2 days (lower grouping) at 33.5°C.

SSL interactions with a number of genes (*CCW12*, *CNB1*, *ECM30*, *KRE1*, *MNN10*, *ROM2*, *SMI1 *and *TOR1*) that have functional links to cell wall organization and biogenesis was consistent with the calcofluor white and ethanol sensitivity of the *tra1*_*SRR*3413 _strain [9]. We thus investigated the extent to which the temperature sensitivity of the *tra1*_*SRR*3413 _strain results from cell wall destabilization by examining if it is suppressed by growth in media containing 1 M sorbitol. As shown in Figure 2, sorbitol partially, but not completely, suppressed the temperature sensitive growth at 37°C of this strain (compare relative growth of *tra1*_*SRR *_and *TRA1 *in SC with sorbitol, lower panel, and without sorbitol, upper panel), indicating that defects in cell wall function contribute to but are not exclusively responsible for the temperature sensitivity. We also examined if some of the SSL interactions were largely due to cell wall instability. *mdm34Δ0 tra1*_*SRR*3413_, *swc3Δ0 tra1*_*SRR*3413_, *mon2Δ0 tra1*_*SRR*3413 _and *cog5Δ0 tra1*_*SRR*3413 _were examined as representative of mitochondrial, chromosomal and membrane sorting groups. As shown in Figure 3, growth inhibition of the *mdm34Δ0 tra1*_*SRR*3413 _strain (at 33.5°C for this experiment in contrast to 37°C for Figure 2) was partially rescued by 1 M sorbitol. Slight suppression was seen for the *cog5Δ0 tra1*_*SRR*3413 _strain, while growth of the *swc3Δ0 tra1*_*SRR*3413 _and *mon2Δ0 tra1*_*SRR*3413 _strains was relatively unchanged by sorbitol. The strain dependent differences in the ability of sorbitol to suppress SSL effects further establish that Tra1 has roles in multiple processes.

### Connection of TRA1 to cellular stress

**Figure 4:**
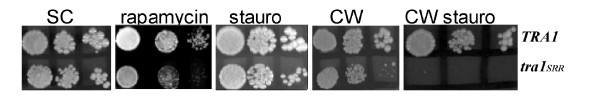
**Growth of the *tra1*_*SRR*3413 _strain under conditions of cellular stress**. Yeast strains BY7092 (*TRA1*) and CY2222 (*tra1*_*SRR*3413_) were grown overnight to saturation in YPD at 30°C. 5 μl of 10-fold serial dilutions were spotted onto synthetic complete media containing 2% glucose (SC) or with the addition of 2 nM rapamycin, 2 μg/ml staurosporine (stauro), 7.5 μg/ml calcofluor white (CW), or 2 μg/ml staurosporine plus 7.5 μg/ml calcofluor white (CW stauro). Cells were grown for 3 days on SC and 5 days on selective plates, at 30°C.

We were intrigued by the finding that amongst the genes showing SSL interactions with *tra1*_*SRR*3413_, 30 are annotated to stress response or autophagy (see Table 1). This is an underestimate as, for example, Swi4 [18], Rps27b [19], Caf40 [20], Vps1 [21] and Stp1 [22] have been implicated in stress response but are not directly annotated as such. Some of the stress response genes include key signaling and regulatory molecules such as Tor1, Msk1, Cnb1, and Rom2; others have functions in protein turnover (Ubi4 and Rpn4), organization of the cytoskeleton (Tpm1 and Bem4) and DNA repair (Rad52, Ckb2, Doa1). To further examine the link between Tra1 and cellular stress response we tested the sensitivity of the *tra1*_*SRR*3413 _strain to rapamycin and to staurosporine. As shown in Figure 4, the *tra1*_*SRR*3413 _strain was partially sensitive to rapamycin, and to a lesser extent staurosporine. As staurosporine results in cell wall instability through its action on Protein Kinase C [23,24], we also examined the *tra1*_*SRR*3413 _strain for sensitivity to the combination of calcofluor white and staurosporine. The *tra1*_*SRR*3413 _strain was extremely sensitive to calcofluor white in the presence of staurosporine, consistent with a role for Tra1 in events required for cell wall integrity.

### Relationship of tra1_*SRR*3413 _to other mutations in SAGA/SLIK and NuA4 components

**Figure 5:**
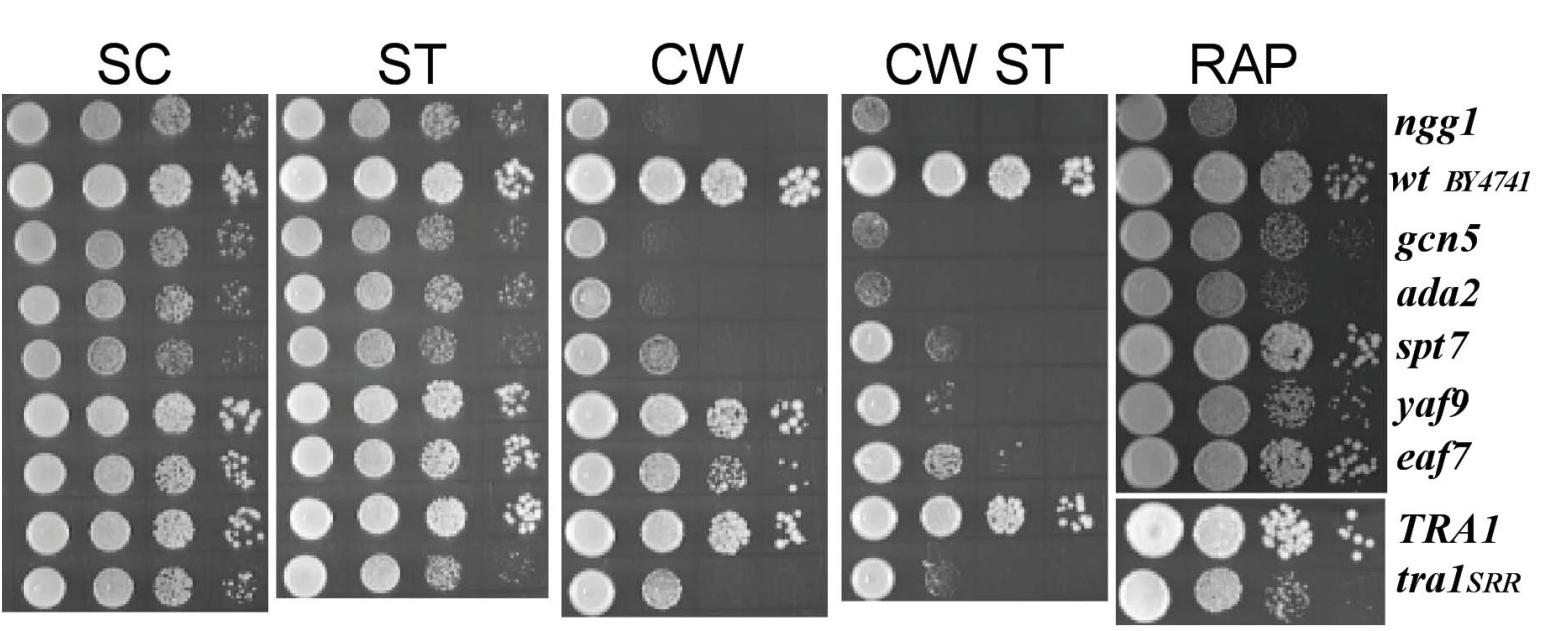
**Phenotypic comparison of *tra1*_*SRR*3413 _with deletions of SAGA/SLIK and NuA4 components**. Yeast strains BY3534 (*ngg1Δ0*), BY4741 (wild-type background for deletion strains), BY7285 (*gcn5Δ0*), BY4282 (*adaΔ0*), BY3281 (*spt7Δ0*), BY4240 (*yaf9Δ0*), BY2940 (*eaf7Δ0*), BY7092 (*TRA1*) and CY2222 (*tra1*_*SRR*3413_) were grown overnight to saturation in YPD at 30°C. Cells were diluted to approximately 2000 cells per μl and 5 μl of 10-fold serial dilutions spotted on synthetic complete media containing 2% glucose (SC), with 2 μg/ml staurosporine (ST), 7.5 μg/ml calcofluor white (CW), or 2 μg/ml staurosporine plus 7.5 μg/ml calcofluor white (CW ST) or YPD with 2 nM rapamycin (RAP). SC and ST were grown for 2 days at 30°C, CW and CW ST for 3 days, and RAP for 4 days. For the rapamycin image, *TRA1 *and *tra1*_*SRR *_were grown on a separate plate.

Bruno *et al*. [25] have shown that deletion of the Ada2 homologue in *C. albicans *results in sensitivity to cell wall destabilizing agents. In addition, both SAGA/SLIK and NuA4 have been suggested to be involved in stress response based upon the transcription profiles of deletion strains [26,27]. As some of the genetic interactions and phenotypes of the *tra1*_*SRR*3413 _strain may result from a SAGA/SLIK or NuA4-dependent inability to induce stress response genes, we analyzed staurosporine, calcofluor white, calcofluor white plus staurosporine and rapamycin sensitivity in strains deleted for additional components of these complexes (Figure 5). The effect of *tra1*_*SRR*3413 _under each of these conditions is similar to, though slightly less severe than, that seen upon deletion of the *ada *genes (*ada2*, *ngg1/ada3 *and *gcn5/ada4*) of the SAGA/SLIK complexes. Interestingly, despite the fact that deletion of *spt7 *results in disruption of the complexes, *spt7Δ0 *results in less of an effect on rapamycin and calcofluor white than deletion of the *ada *genes, suggesting a potential role for an independent Ada complex [28]. This similarity between the *ada *genes and *tra1*_*SRR*3413 _is also consistent with our previous finding that the expression profile in the *tra1*_*SRR*3413 _strain most closely resembled deletion of *ada2 *[9]. Less similarity was seen in the phenotypes with NuA4 components though deletion of *yaf9 *and *eaf7 *did lead to reduced growth in calcofluor white plus staurosporine containing media and deletion of *yaf9 *resulted in a slight reduction of growth in media containing rapamycin.

### GFP-tagged Tra1 localizes to nucleus

**Figure 6:**
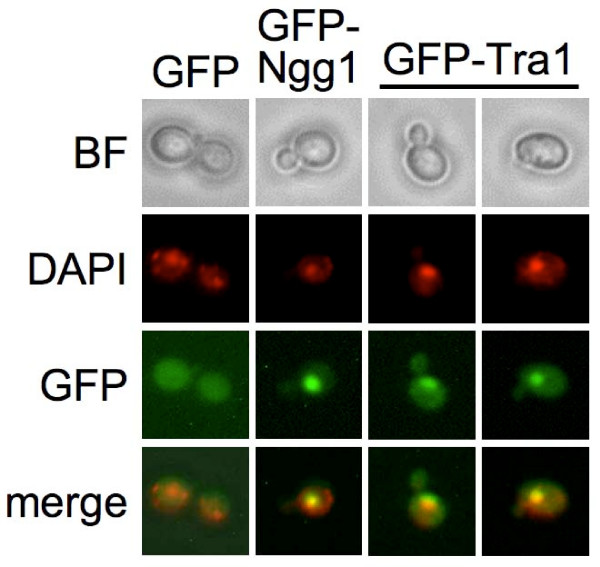
**GFP-Tra1 is found predominately in the nucleus**. Yeast strain BY4741, expressing either GFP, GFP-Ngg1 or GFP-Tra1 from a *URA3*-centromeric plasmid was grown in synthetic complete media, stained with DAPI and examined by fluorescence microscopy. BF, bright field.

The requirement for an intact C-terminal FATC domain [1] has meant that Tra1 localization has not been tested in genome-wide screens. Localization of Tra1 to sites outside the nucleus potentially could account for the broad range of genetic interactions seen for the molecule. To examine cellular localization, we engineered an N-terminally GFP-tagged allele of *TRA1 *that is functional as determined by plasmid shuffling assays (not shown). As shown in Figure 6, the pattern of GFP-Tra1 distribution was very similar to that of the SAGA/SLIK component Ngg1/Ada3 and coincided with DAPI staining within the nucleus (Figure 6). A low level of fluorescence was apparent throughout the cell, making it difficult to fully exclude that lesser amounts exist in other compartments; however, GFP-Tra1 was not in foci other than the nucleus.

## Discussion

Identification of the SSL interactions for the *tra1*_*SRR*3413 _allele has provided insights into the function of this molecule. The genes identified link Tra1 to many cellular functions including membrane sorting/protein trafficking, cell wall biogenesis/function, RNA processing, gene expression and mitochondrial function. The number of genetic interactions identified for *tra1*_*SRR*3413 _is likely due to its function in two key cellular regulatory complexes, SAGA/SLIK and NuA4.

The finding that the SSL profile for *tra1*_*SRR*3413 _does not share greater similarity with mutations of other SAGA/SLIK and NuA4 components agrees with our previous observations that these strains differ in their transcription profiles, effects on telomere length and sensitivity to the DNA damaging agent methylmethanesulfonate [9]. These differences may arise from the integrative effects of disturbing both complexes and/or the possibility that Tra1 has a unique function. While differences are apparent, we do note that if the cluster analysis is performed in the absence of the *arl1Δ0*, *arl3Δ0*, *gyp1Δ0*, *ric1Δ0*, *ypt6Δ0 *and *swf1Δ0 *group, the SSL profile for *tra1*_*SRR*3413 _clusters most closely with SAGA/SLIK and NuA4 components (not shown). In addition, as seen by the common sensitivity to ethanol and calcofluor white plus staurosporine, several of the *tra1*_*SRR*3413 _phenotypes are similar to deletions of the *ada *genes.

### Nuclear function

SAGA/SLIK and NuA4 are nuclear complexes and indeed we have found Tra1 to be predominately, if not exclusively, localized in the nucleus. As an essential component of two histone-modifying complexes, it is not surprising that approximately 40 of the SSL interactions are with genes annotated principally as having roles in nuclear function or gene expression. Some of these genes (for example: *pub1*, *pml39 *and *sky1*) are involved with RNA processing, export or stability perhaps reflecting a role for the SAGA complex at the nuclear pore [29]. Another clear subset of SSL interactions includes genes of the SWR1 complex and *htz1*. In this case the relationship with *tra1*_*SRR*3413 _almost certainly reflects their combined importance in determining chromatin structure and their resulting influence on transcription and/or DNA repair. Supporting this argument, SSL interactions have been observed between many components of SAGA and NuA4 with *htz1 *and SWR1 complex genes [30]. Similarly the interaction of *tra1*_*SRR*3413 _with *rad52 *may relate to the function of the NuA4 complex in DNA repair.

### Membrane processes

The association of SAGA/SLIK and NuA4 components with membrane processes has been noted previously. Gcn5 and Spt20 are required for the unfolded protein response [31,32]. A strain with deletion of *eaf1/vid21 *(Vacuolar Import Degradation) was identified in a screen for defects in sorting of carboxypeptidase Y to the vacuole [33] and results in sensitivity to ethanol [34]. In addition, Eaf1 interacts with Vac8, which is required for several aspects of vacuolar function [35]. *eaf3*, *eaf5*, *eaf7*, *eaf1/vid21 *and *yaf9 *were identified as Opi^- ^mutants that result in overproduction of inositol, though not through direct repression of *INO1 *[36]. Mitchell et al. [15] have observed SSL interactions of nonessential NuA4 components with proteins involved in membrane sorting/protein trafficking. They find that deletion the NuA4 component genes leads to defects in vacuolar morphology and similar to what we suggest for the phenotypic effects of *tra1*_*SRR*3413_, predict that the vacuolar defects arise from changes in gene expression.

### Mitochondrial function

Fifteen genes annotated to have links to mitochondrial function were identified in the screen. This may relate to the possible involvement of the SLIK complex in the retrograde response pathway [37]. Interestingly this pathway links mitochondrial metabolism with stress response, signaling the expression of genes that provide required biosynthetic precursors during mitochondrial dysfunction, which results in the loss of tricarboxylic acid cycle activity [38].

### Stress response

Included in the *tra1*_*SRR*3413 _SSL genes are greater than 30 related to cellular stress or stress response. Genetic interactions for *tra1*_*SRR*3413 _with molecules related to cellular stress is consistent with the transcription profiles of strains containing deletions of SAGA/SLIK and NuA4 components [26,27] as well as the calcofluor white, ethanol, rapamycin and temperature sensitivity of the *tra1*_*SRR*3413 _strain. An aspect of the involvement of Tra1 in stress likely includes a role in the cell wall integrity response pathway as indicated by the calcofluor white sensitivity and interactions of the *tra1*_*SRR*3413 _SSL profile with molecules required for membrane sorting/protein trafficking and cell wall components (for example, Kre1 and Ccw12). The processes identified with Tra1 in many ways resemble those seen with Tor1, which as well as its initially defined role in integrating nutrient status with growth, has been genetically and functionally linked to membrane sorting/protein trafficking, cytoskeletal events, microautophagy and to cell well integrity [39-43].

The simplest interpretation of a possible link between Tra1 and stress is to suggest that Tra1 is involved in the expression of stress response genes or genes whose lack of expression results in stress. Consistent with this, in YPD media seventeen genes with an annotation or description indicative of an involvement in stress response have decreased expression of two-fold or greater in a *tra1*_*SRR*3413 _background ([9], Table 2). Many of these genes function in the response to oxidative stress but their exact role in determining phenotype is likely complex given that the *tra1*_*SRR*3413 _strain is not sensitive to the oxidizing agent tert-butylhydroperoxide and genes typically elevated in response to stress are not found induced in the *tra1*_*SRR*3413 _strain.

**Table 2:** Stress related genes down regulated in a *tra1*_*SRR*3413 _background.

**Gene**	**Fold-decrease**^1^	**Description**^2^
*AAD4*	11	Putative aryl-alcohol dehydrogenase. Involved in oxidative stress response.
*TSA2*	6.5	Stress induced thioredoxin peroxidase.
*CCS1*	5.5	Copper chaperone for superoxide dismutase Sod1. Involved in oxidative stress protection.
*AAD6*	4.4	Putative aryl-alcohol dehydrogenase. Involved in oxidative stress response.
*GPX2*	3.6	Phospholipid hydroperoxide glutathione peroxidase. Protects cells from peroxides.
*SSA4*	3.3	Heat shock protein that is highly induced upon stress. Role in SRP-dependent cotranslational protein-membrane targeting.
*GRX2*	3.1	Glutaredoxin, thioltransferase, glutathione-dependent disulfide oxidoreductase. Maintains redox state of target proteins.
*RHR2*	3.0	DL-glycerol-3-phosphatase. Induced by anaerobic and osmotic stress.
*ALD2*	2.4	Aldehyde dehydrogenase. Stress induced.
*TRX2*	2.4	Thioredoxin isoenzyme. Protects cells against oxidative stress.
*GRE2*	2.4	3-methylbutanal reductase and methylglyoxal reductase. Stress induced.
*SRX1*	2.4	Sulfiredoxin. Contributes to oxidative stress resistance.
*ALD3*	2.3	Cytoplasmic aldehyde dehydrogenase. Stress induced.
*LSP1*	2.3	Component of eisosomes. Null mutants show activation of Pkc1p/Ypk1p stress pathways.
*PST2*	2.2	Similarity to flavodoxin-like proteins. Induced by oxidative stress.
*CMK1*	2.2	Calmodulin-dependent protein kinase. May play a role in stress response.
*DAK1*	2.0	Dihydroxyacetone kinase. Involved in stress adaptation.

^1 ^Decrease in gene expression from microarray analysis [9], *tra1*_*SRR*3413 _versus *TRA1*

^2 ^Modified from the Saccharomyces Genome Database

We previously demonstrated that *tra1*_*SRR*3413 _results in a generation dependent telomere shortening that is not characteristic of other SAGA/SLIK or NuA4 components. Fifteen of the genes with SSL interactions with *tra1*_*SRR*3413 _also show telomere shortening [44]. In many cases direct telomeric functions for these molecules have not been described but like *tra1*_*SRR*3413_, they display slow growth in response to ethanol, calcofluor white or rapamycin. This suggests the possibility that shortened telomeres in the *tra1*_*SRR*3413 _strain is the result of a similar indirect mechanism rather than direct action at the telomere.

## Conclusion

Through the identification of synthetic sick/lethal interactions with *tra1*_*SRR*3413 _we have demonstrated a genetic association of Tra1 not only with nuclear processes but also with membrane events and mitochondrial function. The identity of the SSL genes also connects Tra1 with cellular stress, a result confirmed by the sensitivity of the *tra1*_*SRR*3413 _strain to a variety of conditions that result in a stress response. The transcription profile and SSL interactions indicate that the functions of Tra1 can not simply be ascribed individually to either SAGA/SLIK or NuA4 complexes. However, the finding that many patterns of the *tra1*_*SRR*3413 _phenotype resemble those seen with deletions of the Ada components of the SAGA/SLIK complex points toward a role for the PI3K domain of Tra1 in regulating the activity of the Ada molecules.

## Methods

### Yeast strains and growth

Yeast strains BY3534 (*ngg1Δ0*), BY7285 (*gcn5Δ0*), BY4282 (*adaΔ0*), BY3281 (*spt7Δ0*), BY4240 (*yaf9Δ0*), BY2940 (*eaf7Δ0*) are derivatives of BY4741 (*MATa his3Δ0, leu2Δ0 met1Δ0 ura3Δ0*; [45]) and were purchased from Open Biosystems. Yeast strain CY2222 is a derivative of BY7092 (*MATα can1Δ::STE2pr-SpHIS5 lyp1Δhis3Δ1 leu2Δ0 ura3Δ0 met10 LYS2+) *that has been gene replaced with *tra1*_*SRR*3413 _and selected for through the placement of Tn10LUK at the downstream *Bst*BI site. To ensure that this integration did not hamper expression of YHR100C, a 2035 base pair *Eco*RI-*Hind*III fragment encompassing this gene was integrated after cloning into the *LEU2 *integrating vector YIplac128 [46] and digestion with *Msc*I. Strains containing disruptions of individual genes and double mutants with *tra1*_*SRR*3413 _were obtained by tetrad dissection of diploids generated from the SGA analysis. Strains were spotted in ten-fold serial dilutions on synthetic complete media containing 2 nM rapamycin (LC Laboratories, Woburn Ma), 7.5 μg/ml calcofluor white (Sigma-Aldrich Canada, Oakville Ontario), 2 μg/ml staurosporine (LC Laboratories), calcofluor white plus staurosporine, or 1.0 M sorbitol. Growth was also compared on synthetic complete media containing 1% potassium acetate and 0.05% glucose as the carbon source.

### GFP fusion protein and microscopy

A *URA3 *centromeric plasmid that allowed expression of GFP fusions was engineered by inserting a *Bam*HI-*Not*I fragment of the PCR product synthesized using oligonucleotides 5'-ATGCGGATCCACAATGGTGAGCAAGGGCGAGG-3 and 5'-TTTTCCTTTTCCGGCCGCCTTGTACAGCTCGTCCATG-3' as primers and pEGFP-N1 (Clontech Laboratories, Inc.) as template to replace the tags of YCpDed-TAP-Flag [9]. *TRA1 *and *NGG1 *were inserted into this vector as *Not*I-*Sst*I fragments from YCpDed-TAP-Flag-*TRA1 *(9) and YCpDed-myc-*NGG1 *[47], respectively. Fluorescence microscopy was performed as described by Brachat *et al*. [48] with minor modification. Yeast expressing GFP-tagged proteins were grown in SC media lacking uracil at 30°C. At A_600_~0.8, 4',6-diamidino-2-phenylindole (DAPI) was added to a final concentration of 5 μg/ml and the cells incubated at 30°C for an additional 1–2 hr. Cells from 1 mL of the culture were pelleted, resuspended in 100 μL SC media, immobilized between a microscope slide and cover slip in SC media containing 0.7% agarose and observed using a Zeiss Axiovert 25 fluorescent microscope under 400× magnification. Images were captured, auto-equalized, colorized and merged using Northern Elite software (Empix Imaging, Inc.).

### Systematic genetic-array analysis

SGA analysis with yeast strain CY2222 was performed as described by Tong and Boone [49]. Each strain was pinned in quadruplicate with strains analyzed on synthetic media at 26°C, 34°C and 36°C. Diploids of those strains showing slow growth at all temperatures were sporulated and subjected to tetrad dissection on YPD plates. When viable, the growth of single and double disruptions of the relevant gene were compared to CY2222 on YPD plates at 30°C and synthetic complete plates at 33.5°C. Agglomerative hierarchical clustering based on the average linkage of uncentered correlations using CLUSTER 3.0 software [50] was performed on the profile obtained with the data sets of Tong et al. [11], Measday et al. [12], Reguly et al. [13], Pan et al. [14] and Mitchell et al. [15], as compiled by Mitchell et al. [15]. SSL interactions, as listed in the Saccharomyces Genome Database (March 2008), of additional components of SAGA/SLIK and NuA4 complexes were also included in the analysis.

## Authors' contributions

SMTH carried out microscopy, data analysis and co-wrote the manuscript with CJB. JG performed the SGA analysis under the supervision of BA. CJB performed the analysis of individual strains. All authors have read and approved the final manuscript.
